# Caries-Free Prevalence among Schoolchildren in Malaysia—Time-Series Analysis of Trends and Projections from 1996 to 2030

**DOI:** 10.3390/children10020264

**Published:** 2023-01-31

**Authors:** Lokman Najihah, Wan Zakiyatussariroh Wan Husin, Tengku Mardhiah Tengku Jalal, Jamaludin Marhazlinda

**Affiliations:** 1 Department of Community Oral Health & Clinical Prevention, Faculty of Dentistry, Universiti Malaya, Kuala Lumpur 50603, Malaysia; 2 Mathematical Sciences Studies, College of Computing, Informatics and Media, Universiti Teknologi MARA Cawangan Kelantan, Machang 18500, Malaysia

**Keywords:** caries-free prevalence, schoolchildren, time-series, trend, projection

## Abstract

This study assessed caries-free prevalence trends over two decades from 1996 to 2019 and projected the caries-free prevalence from 2020 to 2030 among schoolchildren in Malaysia. The study consisted of secondary data analysis of caries-free prevalence from 1996 to 2019 in six-, twelve- and sixteen-year-old schoolchildren obtained from Health Information Management System (HIMS) reports. Three time-series models were compared: double exponential smoothing (DES), autoregressive integrated moving average (ARIMA) and the error, trend and seasonal (ETS) model, and the best model with the smallest error was chosen for univariate projection of caries-free prevalence of each age group until 2030. An upward trend of caries-free prevalence was observed for all age groups over the years. Caries-free prevalence was projected to increase with a different increment in each age group for the next decade, with a slightly damped trend noted in 16-year-old schoolchildren. Of all the age groups, the caries-free prevalence trend and projection demonstrated to be highest in 12-year-olds, followed by 16-year-olds, while 6-year-old schoolchildren revealed the lowest caries-free prevalence over three decades. The 16-year-old schoolchildren demonstrated the smallest predicted increment in caries-free prevalence. Future work can explore multivariate projections. Meanwhile, more resources and interventions could prioritise all age groups.

## 1. Introduction

Dental caries is recognised as a significant oral health problem, with 34.1% of the world population suffering untreated caries in permanent teeth and 7.8% of children suffering untreated caries in deciduous teeth [1]. It originates from microbiologic shifts within dental plaque and causes dissolution of the mineral structure on hard dental tissues [2,3]. The disease progresses very slowly and can be stopped or reversed in the early stages, or it can also progress and lead to tooth loss [4,5].

In children, dental caries is the most prevalent oral disease [6]. The impacts of dental caries on children include difficulties in chewing, speech impairment, reduced school performance, malnutrition, social interaction issues, such as smiling, and psychological problems, such as irritation and difficulties with sleeping [7,8]. In addition, reduced nutritional intake due to loss of appetite and pain leads to reduced weight gain and abnormal cognitive development among children [9,10].

However, although caries significantly impacts health, caries is mainly preventable [11]. Tackling caries before the disease starts or at an early stage improves caries-free occurrence and helps to combat other severe and expensive non-communicable illnesses. Hence, an emphasis on maintaining oral health at the individual level would lead to a shift in dentistry practice from restorative to preventive-oriented strategies, where the purpose is directed toward a "caries-free future" before caries develop [12]. 

In Malaysia, a national survey conducted once every ten years reported a decreasing trend in the sum number of decayed, missing due to caries and filled teeth in permanent teeth (DMFT) score among 12-year-old schoolchildren from 1.6 (1997) to 1.1 (2007) and 0.8 (2017). The prevalence of caries also decreased over three decades from 60% (1997) to 40% (2007) and 30% (2017) [13]. Among pre-school children five years of age, the reported decayed and filled primary teeth (dft) score showed a slow reduction in three decades, with dft scores of 5.8 (1995), 5.5 (2005) and 4.8 (2015). Meanwhile, the prevalence of caries also decreased from 87.1% to 76.2% and 71.3% over the same study period. However, even though the caries trend was declining, the caries-free prevalence was far from the target of the National Oral Health Plan to achieve a 50% caries-free score among six-year-old children by 2020 [14].

Simultaneously, data on caries prevalence, severity and patterns among the population and projected changes are precious in planning dentistry’s future [15]. Although it is a common practice in healthcare studies to project future disease incidence or events, such as the studies performed by Ahmar and Boj [16] and Xi, et al. [17], application of projection analysis is still limited in dental studies and not yet widely used in Malaysia, except for one study that was performed by Ismail, et al. [18] that forecast the treatment needs among periodontal patients in International Islamic University Malaysia (IIUM) Dental Clinic. Additionally, only a few studies were performed globally on distribution and projection of dental caries, with the majority of the studies having been conducted in more developed countries, such as China and Germany, where the caries-free prevalence was better than in low- and middle-income countries, such as Malaysia [19,20]. Furthermore, most projection studies only used a single model for projection [21,22].

As such, the present pioneering study assessed dental-caries-free prevalence trends from 1996 to 2018 in 6-year-old schoolchildren and from 1996 to 2019 for 12- and 16-year-old schoolchildren. It also projected the caries-free prevalence for these three age groups in the next decade from 2019 or 2020 until 2030. It was projected by 2030 to measure the progress of oral health status in Malaysia toward attaining the FDI World Dental Vision goals to deliver optimal and comprehensive oral healthcare for all by 2030 [23]. The focus was on caries-free prevalence instead of caries prevalence to promote the prevention-based strategy and progress toward a ‘cavity-free’ future. Furthermore, the shift from disease management to preventive care in the long term could be more cost-effective and improve disability-adjusted life years (DALY) due to oral diseases.

## 2. Materials and Methods

### 2.1. Study Design and Participants, Data Source and Description

The study consisted of secondary data analysis using data on dental-caries-free prevalence among schoolchildren attending government schools in Malaysia retrieved from Health Information Management System (HIMS) reports. These children received incremental dental care through the school dental services rendered by the Oral Health Division, Ministry of Health. All the regular reports regarding healthcare services, including oral health, are collected at the operational level (schools and clinics) and gathered nationwide through a web-based report system known as HIMS, a database for healthcare delivery programs.

Dental-caries-free prevalence was defined as the presence or absence of caries. Therefore, absence indicated a caries-free presence, which was the outcome variable in this study. The researchers retrieved all the data about dental-caries-free prevalence among 6-, 12- and 16-year-old schoolchildren from the HIMS report. However, there was no sample size calculation since, in time-series analysis, the required datapoints should be as many as possible to accurately estimate a model because time-series analysis entirely relies on the previous behaviour of the data [24,25]. As a result, the extracted data consist of 23 years of caries-prevalence data from 1996 to 2018 for six-year-old schoolchildren. Meanwhile, among 12- and 16-year-old schoolchildren, the extracted data consist of 24 years of caries-free prevalence data from 1996 to 2019. The Medical Ethics Committee, Faculty of Dentistry, the University of Malaya (DF CO2009/0035 (L) approved the research.

### 2.2. Data Analysis

In this study, R software version 4.2.2 was used to analyse univariate time series data using the error trend and seasonal (ETS) mode. Meanwhile, IBM SPSS version 25 was used to analyse univariate time series data using the double exponential smoothing (DES) and autoregressive integrated moving average (ARIMA) models. During data exploration and cleaning, no missing value was observed in the study’s outcome. Three different forecasting models, DES, ARIMA and ETS model in time series, were compared, and the model with the lowest error was chosen to provide the best and most accurate projection of dental-caries-free prevalence for each age group. The best ARIMA and ETS model were selected using automatic selection according to minimal Akaike′s information criterion (AICc) score. The model does not require the data to be stationarity prior to automatic selection. During the automatic selection process, the model variables are auto-transformed using differencing, a square root transformation or a natural log transformation when necessary [26].

DES was chosen because this smoothing technique is useful for series exhibiting linear trend characteristics with no seasonality [27,28]. Meanwhile, ARIMA applies to all fields and can describe and handle autocorrelated data [24,29]. ETS is a time series forecasting method for exponential smoothing, consisting of measurement equations that describe the observed and hidden components (level, trend, seasonal) that change over time. ETS is also suitable for a data series with or without linear, seasonal or damped trends [24]. As the requirements of these models work well with the behaviour patterns and characteristics of caries-free prevalence data, these models (DES, ETS and ARIMA) were compared and only the best model with minimal forecast errors was selected for projection of caries-free prevalence for each age group. 

Initially, the data were divided into two parts: in-sample data and out-sample data. The in-sample data were used to estimate the parameters, and the out-sample data were used to evaluate model accuracy. The size of the out-sample data was 20% of the whole dataset, as [24] suggested. In this study, the estimation part or in-sample data covers the first 18 years of the time horizon for six-year-old schoolchildren, from 1996 until 2013. The following years (2014 to 2018) were considered out-sample data or evaluation parts. Meanwhile, for 12- and 16-year-old schoolchildren, the estimation covers the first 19 years of the time horizon, which lasted from 1996 to 2014. Then, from 2015 to 2019, the remaining years were considered the evaluation part.

The evaluations of the models were divided into two phases. The first phase was based on caries-free in-sample fitting evaluation and out-of-sample or forecast evaluation for each model. The out-sample evaluation was based on a five-step-ahead forecast generated using each model. Then, forecast errors were computed by comparing the forecasts with the actual data. Meanwhile, the one-step-ahead cross-validation technique, also known as rolling forecasting origin, was employed in the second phase. This approach uses many different training sets (n = 5), each containing one more observation than the previous one. Then, the forecast accuracy measures are calculated on each test set and the results are averaged across all test sets.

Subsequently, the performance of the models (DES, ARIMA and ETS) was evaluated, and the best time series model for each age group was selected according to the lowest errors in mean absolute error (MAE), mean absolute percentage error (MAPE), mean square error (MSE), root mean square error (RMSE) and mean absolute deviation error (MAD). However, all these errors served different purposes and no single accuracy measure was suitable for all types of data and models. In addition, numerous studies provide evidence that an excellent method in one criterion is not necessarily suitable for another [30,31]. Thus, it is imperative to use several error measures to evaluate the model′s accuracy. Therefore, the best model was chosen based on the most significant number of error measures with a minimal score. 

## 3. Results

In this trend and projection study, the estimated total number of schoolchildren attendance was 10,515,676 six-year-olds from 1996 to 2018, 11,127,663 12-year-old schoolchildren and 7,820,688 16-year-old schoolchildren from 1996 to 2019. As the incremental school program only involved students from a government school, only 70–80% of the children in Malaysia who attended the government school were qualified for this program. However, the population covered in this study varied depending on school coverage over these years. School coverage for primary and secondary schoolchildren ranged from 81.75% to 97.3% in primary school and from 56.86% to 91.0% in secondary school.

### 3.1. Trends of Caries-Free Prevalence among 6-, 12- and 16-Year-Old Schoolchildren in Malaysia from 1996 to 2019

**Figure 1:**
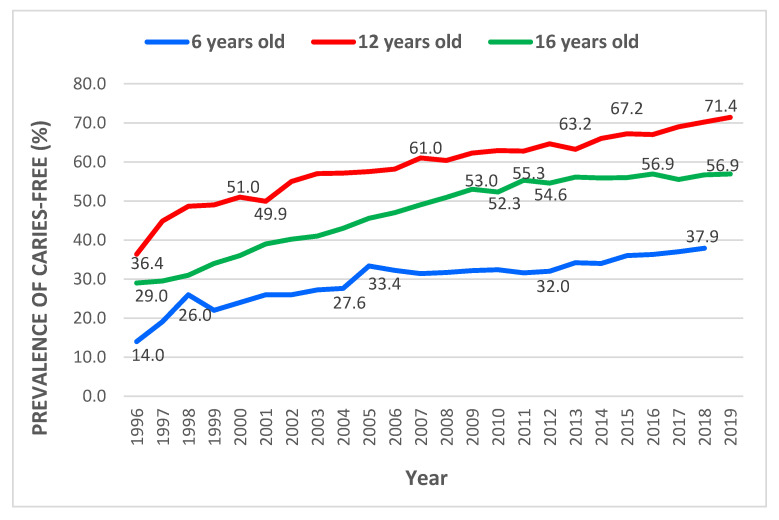
Trend of caries-free prevalence among six-year-old schoolchildren (1996–2018) and among 12- and 16-year-old schoolchildren (1996–2019) in Malaysia.

Figure 1 shows the trend of caries-free prevalence among 6-, 12- and 16-year-old schoolchildren in Malaysia. Over 23 years of the study horizon, caries-free prevalence among 6-year-old children in Malaysia showed an upward trend, with two significant spikes recorded in 1998 and 2005. The lowest prevalence recorded was in 1996, with a 14.0% caries-free rate that rose to 26.0% in 1998 and dropped a year later to 22.0%. After six years, this pattern repeated: the caries-free prevalence spiked to 33.4 % and declined to 32.2% in the subsequent year. Malaysia′s highest caries-free prevalence for six-year-old schoolchildren was 37.9% in 2018. Meanwhile, among 12-year-old schoolchildren, there was also an increasing trend of caries-free prevalence over the 24 years of the study horizon. The lowest caries-free prevalence was observed in 1996 at 36.4%, and the highest was in 2019 at 71.4%. However, during the initial years, the slope of caries-free increment was steeper than in more recent years. The caries-free prevalence rose to 51.0% in 2000 from 36.4% in 1996 and slightly dropped a year later to 49.91%. Then, the trend increased for another six years before declining to 60.4% in 2008 and another drop from 64.0% in 2012 to 63.2% in 2013. The trend of caries-free prevalence then continued to increase, albeit less sharply than in earlier years, with the highest prevalence recorded in 2019 at 71.4%. 

For the 16-year-old children, an increasing trend throughout 24 years with a damped trend from 2011 toward the end of the study period was observed. However, the caries-free prevalence trend among this age group showed no significant changes from 2011 to 2019, with caries-free prevalence improving from 55.3% in 2011 to 56.9% in 2019. The highest prevalence recorded was in 2016 and 2019, at 56.9%. Consistently, over the years, the caries-free prevalence trend was highest among 12-year-olds, followed by 16-year-old schoolchildren, while the lowest was among 6-year-old schoolchildren. The increment of caries-free prevalence over the years was highest among 12-year-olds with a 35% increment, followed by 16-year-olds at 27% and then 6-year-old schoolchildren at 23%.

### 3.2. Selection of the Best Time Series Model for Projecting Caries-Free Prevalence for Each Age Group

From the automatic selection method, among the 6- and 12-year-old groups, ETS (A,Ad,N) was chosen for model comparison. ETS (A, Ad, N) is a model with additive error (A) and an additive damped trend (Ad) but with no seasonality (N). Meanwhile, for 16-year-old children, the chosen model was ETS (A, A, N), a model with additive error and a trend but no seasonality. As for the ARIMA model, the auto-arima function selected ARIMA (0,1,0) with first-order differencing for model comparison in all age groups. These models (DES, ETS and ARIMA) were compared. Based on the performance of DES, ETS and ARIMA on their ability to fit accuracy (in-sample evaluation) and forecast accuracy (out-sample evaluation and cross-validation), the best model with the greatest number of error measures with a minimal score for fitted accuracy, forecast accuracy and cross-validation was identified. Details are shown in Table 1, Table 2 and Table 3, respectively.

For model selection, the best model was selected based on the highest number of error measures with a minimal score. Thus, ARIMA (0,1,0) was the best model in the 6-year-old group with the lowest errors in both forecast accuracy techniques, ranging from 0.40 to 4.18 (Table 2 and Table 3). Meanwhile, for 12-year-olds and 16-year-olds, the best model was DES as it reported the lowest errors in both forecast accuracies compared to other models, with the errors ranging from 0.36 to 1.75 and 0.21 to 3.41, respectively. The model with the lowest error in each error measure is highlighted in Table 1, Table 2 and Table 3. All the selected best fitted models for all the age groups showed minimal errors and MAPE errors of less than 10, which indicates highly accurate forecasts [32]. The Box Ljung statistic for the selected model shows non-significant results with *p*-value = 0.749 (6 years old), *p*-value = 0.352 (12 years old) and *p*-value = 0.480 (16 years old), which indicate that the models are fit and assumption of random errors was satisfied in all models. Therefore, ARIMA (0,1,0) was used to predict the caries-free prevalence among 6-year-old schoolchildren, while the DES model was applied to forecast the caries-free prevalence in 12- and 16-year-old schoolchildren.

### 3.3. Projection of Caries-Free Prevalence in 6-, 12- and 16-Year-Old Schoolchildren until 2030

**Figure 2:**
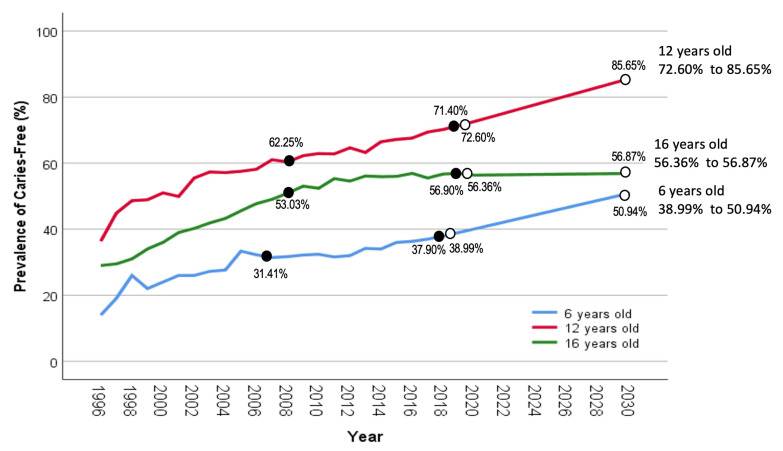
Univariate projection of caries-free prevalence among 6-, 12- and 16-year-old schoolchildren.

The caries-free prevalence was projected to increase over the years, with the highest projected prevalence reported among 12-year-olds, followed by 16-year-old and 6-year-old children, as shown in Figure 2. Among the 6-year-old schoolchildren, it was predicted that caries-free prevalence would rise by 11.95% over 11 years, from 38.99% (2019) to 50.94% (2030), as compared to an increment of only 6.49% over the past 11 years from 31.41% (2007) to 37.90% (2018). As for 12-year-old schoolchildren, the caries-free prevalence was projected to increase by 13.05% over a decade from 72.60% (2020) to 85.65% (2030), which was just slightly greater than the past increment of 9.15% over the same period, from 62.25% (2009) to 71.40% (2019). Further, caries-free prevalence among 16-year-old schoolchildren was forecasted to remain steady in the next ten years, increasing by just 0.51% throughout the years, from 56.36% (2020) to 56.87% (2030), compared to a 3.87% improvement over the same period, from 53.03% (2009) to 56.90% (2019). The results of the projection of caries-free prevalence up to 2030 are summarised in Table 4.

## 4. Discussion

In this study, the ARIMA (0,1,0) model was selected as the most accurate model for projection of caries-free prevalence in the six-year-old group, and the DES model was chosen as the best model for projection of caries-free prevalence in the 12- and 16-year-old groups. However, in future research, the most accurate selected model for future projection of caries-free prevalence in these age groups might be different as the different datapoint lengths would provide different behaviour of data, resulting in a different model for future projection.

Within the limitations of this study, the findings have addressed the study objectives to determine the caries-free prevalence trend over the years among six-, twelve-, and sixteen-year-old schoolchildren in Malaysia. The authors conducted univariate projection of caries-free prevalence of different age groups in the next decade until 2030. Over the past two decades from 1996 to 2019, caries-free prevalence showed an upward trend in all age groups, with the highest prevalence observed among twelve-, followed by sixteen- and six-year-old schoolchildren. However, the highest caries-free prevalence (39%) among six-year-old schoolchildren over the years was far behind their counterparts in Europe and Asia, with 79% and 48% of caries-free prevalence in deciduous teeth, respectively. In comparison, the highest caries-free prevalence values among 12-year-olds (71.4%) and 16-year-olds (56.9%) were higher than in Europe and Asia, with the caries-free prevalence of permanent teeth among the children at 55.9% and 41.2%, respectively [33].

Compared to the other age groups, the low caries-free prevalence among the six-year-old group could be associated with more primary teeth present at this age. Primary teeth enamel was thinner than permanent teeth, with lesser mineral calcium, which may cause the primary teeth to break more easily, hence more caries lesions in this age group [34,35]. Additionally, in Malaysia, despite numerous oral healthcare programmes being provided to young children, utilisation of oral healthcare services is poor, with only 32.3% of preschool children and 17.5% of toddlers receiving primary oral healthcare services in 2019 [36]. This may account for the low caries-free prevalence in the six-year-old group as the services provided were inaccessible in some areas and unevenly distributed across Malaysia [37]. 

Meanwhile, the higher caries-free prevalence among 12-year-old schoolchildren could be associated with newly erupted permanent teeth at this age and, hence, a lower risk of caries. Additionally, it could also reflect the impacts of comprehensive incremental dental care for school-aged children [13]. As of 2019, approximately 94.9% of primary and 91% of secondary school students have participated in this programme since its introduction in 1985 [36]. Furthermore, implementing the school-based fissure sealant programme for primary schoolchildren may be associated with the increase in caries-free prevalence among 12-year-old schoolchildren. Additionally, according to the combined evidence from epidemiological surveys and cross-sectional studies, the reduction in caries cases in Malaysia also related to successful coverage of water fluoridation [38]. As of 2020, it was estimated that 72.8% of the Malaysian population have access to water fluoridation, which also may contribute to the excellent caries-free prevalence in the 12-year-old group [36].

The lower caries-free prevalence in 16-year-olds compared to 12-year-olds could be due to a lack of oral health awareness and the transition from childhood to adulthood. Children started to stay longer hours outside without their parents’ supervision and began undertaking self-made decisions on diet preference and oral hygiene [39]. Additionally, the damped trend and projection of caries-free prevalence in the next decade with slight improvement may symbolise unmet dental needs, especially in distant areas. Therefore, the existing intervention strategy could be revised or modified for this age group to be more relevant. More focus is required on controlling progression of caries in the minority child population, where the disease burden remains exceptionally high [40].

In terms of projection, caries-free prevalence was forecasted to increase steadily in 6- and 12-year-old schoolchildren from 2020 to 2030. However, the findings revealed a worrying future trend of caries-free prevalence among the 16-year-old group. The damped trend toward 2030 may indicate the effects of oral health promotion and prevention strategies delivered to this age group. In Malaysia, oral health education was part of incremental dental care in school dental services provided to schoolchildren throughout their childhood. Nevertheless, the delivery method may play a crucial role in determining the efficiency and effectiveness of oral health messages, especially for this adolescent age group [41]. Therefore, the method and content must be relevant to the age group. For example, dental health talks versus oral health applications may influence adolescents differently. 

On the contrary, although caries-free prevalence among six-year-old schoolchildren was expected to increase, it is still far from achieving the National Oral Health Plan (NOHP) of 50% caries-free among six-year-old children by 2020. Based on the forecasting analysis, the six-year-old group was predicted to achieve this goal by 2030, a ten-year difference from the national target. Thus, there is room for further improvement in the current oral health programmes targeted to this age group. More intense holistic and integrated strategies requiring greater cooperation from the stakeholders, such as pre-schoolers, teachers and caregivers, could be implemented to improve caries-free prevalence, hence preventing any morbidity from oral diseases. Furthermore, as children with early childhood caries had a greater risk of developing future caries in their permanent teeth, an effective preventive strategy in this age group will indirectly improve caries-free prevalence in other age groups as well [42]. 

The main strength of this study is its design as a nationwide study, and the samples represent all schoolchildren aged 6, 12 and 16 years in Malaysia. Therefore, all the samples from the whole of Malaysia with extensive variations in background and oral health experiences could improve the prediction and generalizability of the findings to other developing countries with similar healthcare systems and socioeconomic status. In contrast to many other studies that concentrate on the trend of dental caries, this study focuses on caries prevention by gathering data on the trend and projection of caries-free prevalence. As recommended by FDI Vision 2030, concentrating on disease prevention, early treatment and prevention of early-stage oral disease can ultimately lessen the risk of numerous health disorders, delivering improved health outcomes and providing long-term financial savings [12]. This is also the first study assessing the trend and projection of dental-caries-free prevalence among schoolchildren in Malaysia. However, this was a univariate time-series analysis and should be followed further by multivariate projections to consider potential associated factors if data over time are available.

## 5. Conclusions

Overall, the DES and ARIMA models demonstrated more practicability and accuracy in predicting caries-free prevalence among schoolchildren in Malaysia. There was an increasing trend of caries-free prevalence for the past 20 years for all age groups of schoolchildren, and it was projected to increase in the next ten years among six- and twelve-year-old schoolchildren in Malaysia. However, the caries-free prevalence in 16-year-old schoolchildren was predicted to reach a plateau with insignificant change in the next decade. Of all the age groups, six-year-old schoolchildren appeared to have the lowest caries-free prevalence. Hence, apart from exploring multivariate projections as future work, more interventions and resources could be prioritised for all age groups.

## Figures and Tables

**Table 1:** Models comparison of fitted accuracy.

Error Measures	Error Values								
	6 Years Old			12 Years Old			16 Years Old		
	DES	ETS (A,Ad,N)	ARIMA (0,1,0)	DES	ETS (A,Ad,N)	ARIMA (0,1,0)	DES	ETS (A,A,N)	ARIMA (0,1,0)
MSE	5.73	4.00	6.82	4.70	3.41	5.55	1.29	1.30	1.65
RMSE	2.39	2.00	2.61	2.17	1.85	2.36	1.14	1.14	1.28
MAPE	8.29	6.37	7.48	3.25	2.39	3.24	2.29	2.30	2.45
MAD	2.10	1.46	1.98	1.62	1.16	1.75	0.93	0.93	0.82
ME	0.95	0.29	0.001	0.68	0.35	0.002	0.39	1.53	0.004

[Estimation part: 1996–2013 (6 years old), 1996–2014 (12 and 16 years old)], grey box-model with lowest error measures. Note: DES = double exponential smoothing; ARIMA = autoregressive integrated moving average; ETS = error trend and seasonal; MSE = mean square error; RMSE = root mean square error; MAPE = mean absolute percentage error; MAD = mean absolute deviation error and ME = mean error.

**Table 2:** Models comparison of forecast accuracy.

Error Measures	Error Values								
	6 Years Old			12 Years Old			16 Years Old		
	DES	ETS (A,Ad,N)	ARIMA (0,1,0)	DES	ETS (A,Ad,N)	ARIMA (0,1,0)	DES	ETS (A,A,N)	ARIMA (0,1,0)
MSE	14.92	4.25	2.64	1.69	4.95	6.17	3.41	2.77	19.68
RMSE	3.86	2.06	1.62	1.30	2.22	2.48	1.85	1.66	4.43
MAPE	9.78	4.92	4.18	1.75	2.89	3.35	3.11	2.78	7.06
MAD	3.59	1.82	1.52	1.22	2.02	2.33	1.57	1.75	3.32
ME	3.59	4.66	1.52	1.22	2.01	2.33	1.56	1.75	3.98

[Evaluation part: 2014–2018 (6 years old), 2015–2019 (12 and 16 years)], grey box model with lowest error measures. Note: DES = double exponential smoothing; ETS = error trend and seasonal; ARIMA = autoregressive integrated moving average; MSE = mean square error; RMSE = root mean square error; MAPE = mean absolute percentage error; MAD = mean absolute deviation error and ME = mean error.

**Table 3:** Comparison of forecast accuracy for one-step-ahead cross-validation technique.

Error Measures	Error Values								
	6 Years Old			12 YEARS Old			16 Years Old		
	DES	ETS (A,Ad,N)	ARIMA (0,1,0)	DES	ETS (A,Ad,N)	ARIMA (0,1,0)	DES	ETS (A,A,N)	ARIMA (0,1,0)
MSE	2.03	1.783	0.74	0.36	2.712	0.65	0.92	1.00	2.25
RMSE	1.42	1.33	0.86	0.60	1.64	0.80	0.96	1.00	1.50
MAPE	2.56	2.97	2.12	0.68	2.315	1.04	1.51	1.53	2.08
MAD	0.91	1.10	0.75	0.47	1.55	0.71	0.85	0.86	1.17
ME	0.73	1.10	0.40	0.47	1.61	0.61	0.21	0.27	1.17

[Evaluation part: 2014–2018 (6 years old), 2015–2019 (12 and 16 years old)], grey box model with lowest error measures. Note: DES = double exponential smoothing; ETS = error trend and seasonal; ARIMA = autoregressive integrated moving average; MSE = mean square error; RMSE = root mean square error; MAPE = mean absolute percentage error; MAD; = mean absolute deviation error and ME = mean error.

**Table 4:** Forecasted cases of caries-free among 6-, 12- and 16-year-old schoolchildren over ten-year time period from 2019 to 2030.

Forecasted Years Ahead	Caries-Free Prevalence (95%CI)		
	6 Years Old	12 Years Old	16 Years Old
2019	38.99 (34.03, 43.95)	-	-
2020	40.07 (33.06, 47.09)	72.60 (68.38, 76.82)	56.36 (53.98, 58.74)
2021	41.16 (32.57, 49.75)	73.91 (69.36, 78.45)	56.41 (53.69, 59.13)
2022	42.25 (32.33, 52.16)	75.21 (70.01, 80.42)	56.46 (53.10, 59.83)
2023	43.33 (32.24, 54.42)	76.52 (70.32, 82.72)	56.51 (52.24, 60.79)
2024	44.42 (32.27, 56.57)	77.82 (70.32, 85.32)	56.57 (51.17, 61.96)
2025	45.50 (32.38, 58,63)	79.13 (70.08, 88.18)	56.62 (49.94, 63.31)
2026	46.59 (32.56, 60.62)	80.43 (69.62, 91.24)	56.67 (48.54, 64.80)
2027	47.68 (32.80, 62.55)	81.74 (68.99, 94.48)	56.72 (47.03, 66.41)
2028	48.76 (33.08, 64.45)	83.04 (68.20, 97.88)	56.77 (45.41, 68.13)
2029	49.85 (33.40, 66.30)	84.35 (67.28, 101.42)	56.82 (43.68, 69.96)
2030	50.94 (33.76, 68.12)	85.65 (66.22, 105.08)	56.87 (41.86, 71.88)

## Data Availability

Not applicable.
